# Interaction of Bacteriophage λ with Its *E. coli* Receptor, LamB

**DOI:** 10.3390/v4113162

**Published:** 2012-11-15

**Authors:** Sujoy Chatterjee, Eli Rothenberg

**Affiliations:** Department of Biochemistry and Molecular Pharmacology, NYU Medical School, 550 First Avenue, New York, NY 10016, USA; Email: sujoy.chatterjee@nyumc.org

**Keywords:** bacteriophage, lambda phage, LamB receptor, protein J, single-virus tracking

## Abstract

The initial step of viral infection is the binding of a virus onto the host cell surface. This first viral-host interaction would determine subsequent infection steps and the fate of the entire infection process. A basic understating of the underlining mechanism of initial virus-host binding is a prerequisite for establishing the nature of viral infection. Bacteriophage λ and its host *Escherichia coli* serve as an excellent paradigm for this purpose. λ phages bind to specific receptors, LamB, on the host cell surface during the infection process. The interaction of bacteriophage λ with the LamB receptor has been the topic of many studies, resulting in wealth of information on the structure, biochemical properties and molecular biology of this system. Recently, imaging studies using fluorescently labeled phages and its receptor unveil the role of spatiotemporal dynamics and divulge the importance of stochasticity from hidden variables in the infection outcomes. The scope of this article is to review the present state of research on the interaction of bacteriophage λ and its *E. coli* receptor, LamB.

## 1. Introduction

Viral infections are initiated through a binding process, which involves a specific interaction between the virus and the host cell surface [1,2,3]. The nature of initial virus host interaction greatly varies amongst different systems, and is mediated by various cell surface receptors, co-receptors and other molecules, depending on the specific virus and host [4,5,6]. Nevertheless, the underlining mechanism of this interaction relies on a diffusion limited viral-receptor finding process. This viral receptor-finding process is crucial for the propagation of the infection process as it determines the fate of infection [4,6]. Here we review the studies of this interaction focused on the *Escherichia coli* bacterium and its virus, bacteriophage λ as a virus-host model system.

Since the seminal discovery of bacteriophage λ, it has drawn paramount interest by the molecular biologists and served as an excellent genetic tool for studying fundamental principles of biology such as gene regulation, DNA replication, homologous and site-specific recombination [7,8,9]. Even today, the λ system continues to yield new insights into its gene regulatory circuits. The best-characterized feature of phage λ biology is the genetic switch that decides whether a phage propagates by cell lysis, or integrates into host genomes to become a prophage [10,11,12]. Other well studied aspects of phage λ biology is the adsorption of the phages and subsequent DNA delivery into the host *E. coli *cell [13,14,15,16]. Phage λ uses the bacterial maltose pore LamB (λ-receptor) for delivery of its genome into the bacterial cell. The interaction between λ and its receptor has been extensively studied both biochemically using purified components and genetically by either mutated phage or bacterial receptor [17,18,19,20,21,22,23,24,25]. However, the quantitative understanding of the dependence of viral target-finding on virus-receptor interactions and cellular architecture came from recent single virus tracking studies [26,27]. In this review we focus on the interaction of phage λ and its receptor LamB. We will summarize previous biochemical and structural studies of phage λ and its receptor (LamB), and provide an overview of a recent study of virus target searching mechanisms at single molecule level. 

## 2. Structure of the LamB Receptor and Interaction with Bacteriophage λ

**Figure 1:**
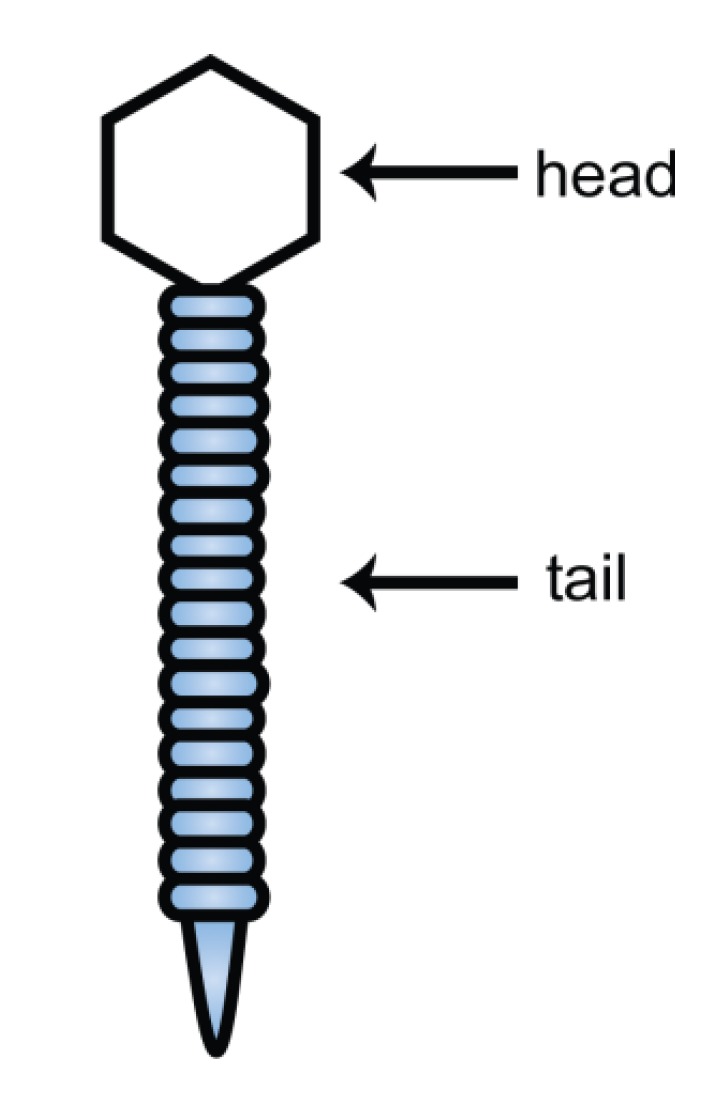
A cartoon representation of λ phage.

Bacteriophage λ is one of the well studied models in molecular biology. Although a vast amount of information is available about the gene regulation network of λ [10,11,12], the quantitative depiction of host cell infection was determined only recently [26]. Bacteriophage λ consists of an icosahedrally symmetric (5,3,2 rotational symmetries) head of diameter 60 nm encapsulating the 48,502 bp double strand DNA molecule and a flexible tail through which the viral DNA expel during infection (Figure 1) [28,29]. Following infection, the invading phage DNA can either replicate within the host, forming new phages and propagate by lysing the host cell (lytic pathway), or it can become a prophage by integrating its DNA into the host chromosome, which then replicate as a part of host chromosome (lysogenic pathway). However, a switch from lysogenic to lytic pathway can be induced where the prophages can replicate independently, assemble the head and tail, forming new viruses and promoting lysis for further propagation [10,11,12]. The *in vivo* head and tail assembly are complex process and beyond the limit of present discussion [29,30]. Early genetic experiments showed that most of the *E. coli* K-12 mutations resistant to λ phage are located in two genetic regions *malA* and *malB* [14,16,21,22]. This *malB* region contains a gene *lamB* whose product, LamB, involves in the λ receptor synthesis, a component of *E. coli* outer membrane. However, a recent study shows that mutant form of bacteriophage λ can target alternative receptor. When phage cI26 (a strictly lytic derivative of phage λ) was cultured with *E. coli* in condition that suppressed the expression of LamB, mutant phage changed their specificity from LamB to a new receptor, OmpF [31]. A combination of four mutants in phage tail protein J were required for targeting this new receptor. It is noteworthy that some host mutations prevented phage from evolving this new function, demonstrating the complexity of interactions in a co-evolving population. The adsorption of λ phages onto bacterial surface is the first step in the infection process [15]. At this stage, phages can either dissociate from the host cell, known as desorption or alternatively bind irreversibly to the host cell [14]. However, once the phage irreversibly bind to the cell surface it triggers a series of poorly understood events and finally delivers its DNA into the bacterial cytoplasm through the channel formed by its tail, leaving the phage protein capsid behind. The tail fibers of bacteriophages are also important to make specific contacts with receptor molecules on the surface of the bacterial cell. The common laboratory strain of bacteriophage λ, so called λ wild type carries a frameshift mutation in *stf* gene relative to Ur-λ, the original isolate. The Ur-λ phages have thin tail fibers which are absent in λ wild type and the Ur-λ has expanded receptor specificity and adsorbs to host cells more rapidly, suggesting the importance of tail fibers [32]. The process between phage adsorption and DNA injection can be sub divided in three steps: ‘lag’, ‘trigger’ and ‘uptake’ [33]. It has been shown that the free tail can itself adsorb to the cell surface, however, the head attachment is required for the lag reaction [9,29,34].

Gram-negative bacteria have two membranes [35]; the outer monolayer is composed of lipopolysaccharide (LPS) as its major lipid, and the inner leaflet contains mostly phosphatidylethanolamine, small amounts of phosphatidylglycerol and cardiolipin [36]. The outer membranes of Gram negative bacteria protect cells from harmful agents by slowing down their penetration while allowing uptake of nutrients [37,38,39]. The major proteins of outer membrane are β‑barrel proteins, which play crucial roles in adhesion and virulence. In Gram negative bacteria β‑barrel proteins are found exclusively in the outer membranes and contributed ~2%–3% of the Gram negative proteome [40]. However, their occurrence is not restricted to Gram negative bacteria and also found in Mycobacteria, mitochondria and chloroplasts [41,42,43,44]. Transmembrane β-barrels also found in several microbial toxins such as heptameric pore-forming α-haemolysin from Staphylococcus aureus, toxin aerolysin and the anthrax-protective antigen [45,46,47]. 

Well characterized constitutive outer membrane proteins are porins. They form a general diffusion pore with a defined exclusion limit for hydrophilic solutes within the outer membrane. In addition to the constitutive porins, the outer membrane may contain porins that are induced only under special growth conditions. The most abundant porins in *Escherichia coli*, the OmpF and OmpC porins, are called general porins, allowing passage of hydrophilic solutes up to a size limit determined by the constrictions of their channels [37]. The biogenesis of β-barrel proteins is complex owing to their intrinsic structure. The insertion of individual β-strands into the hydrophobic core of the bilayer is thermodynamically restricted as they cannot assemble from previously inserted transmembrane segments due to the lack of hydrogen bonds of individual β-strands of β-barrel proteins. Henceforth, membrane insertion must be orchestrated with acquisition of both secondary and tertiary structure to produce the contiguous membrane-spanning barrel held by inter-strand hydrogen bonds [37,38,39]. Furthermore, the porins must also attain their quaternary structure, presumably by associations formed in the membrane. 

**Figure 2:**
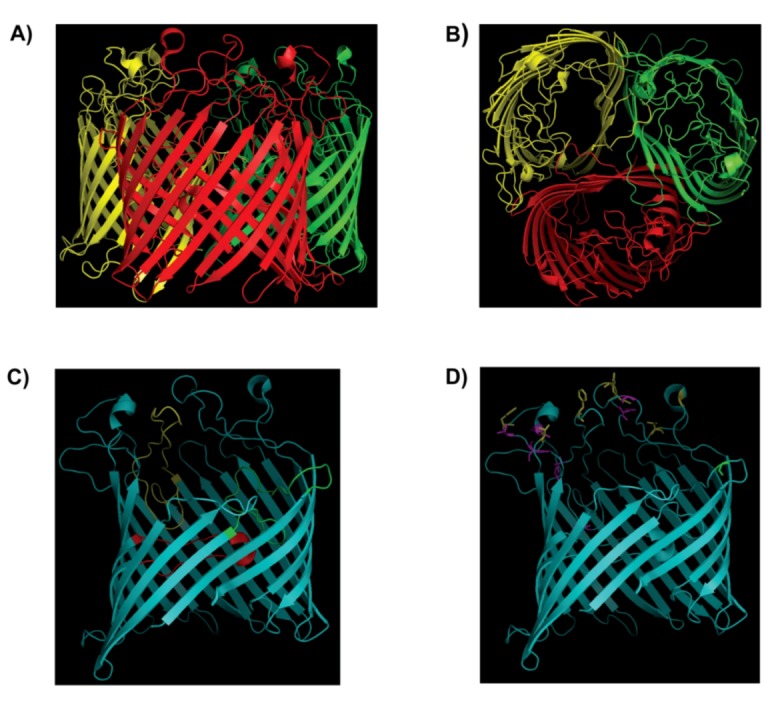
Crystal structure of 18 antiparallelβ-strands barrel trimer LamB. (**A**) Side view and (**B**) Top view. Three monomers are shown by different colors (red, yellow and green). (**C**) Interaction of loops in barrel. Three loops L1 (green), L3 (red) and L6 (yellow) interact with L2 from an adjacent subunit and packed against the inner wall of the barrel. (**D**) The mutation(s) of LamB confer resistance to phage infection. The surface exposed mutant shown in yellow and the others in red. Mutation G18V is known to affect the stability of trimer and shown in green. The Adopted and modified after [54,56].

The LamB protein is a well-characterized example of a porin, termed as a maltoporin, because it is required for growth on limiting concentrations of maltose. The protein coded by gene *lamB* of the maltose operon also serves as receptors for several phages, such as λ, K10 and TP1 [48,49,50]. The molecular weight of LamB is 135.6 kDa, looking like a half-open tulip, formed by 3 identical subunits, each one having a molecular weight of 45.9 kDa [51,52,53]. A major contribution to understanding the molecular basis of the λ phage interaction with LamB receptor has come from determination of the crystal structure of LamB [54]. This study showed that each subunit of the trimeric protein formed by an 18-stranded antiparallel β-barrel, which form a wide channel with a diameter of about 2.5 nm (Figure 2A,B). Loops are found at the end of barrel. Three loops, L1, L3 and L6 (in Figure 2C, they are colored as green, red and yellow respectively) interact with the Loop 2 from an adjacent subunit and packed against the inner wall of the barrel and line the channel. The other loops form a compact structure at the cell surface. Six aromatic residues (Y6, Y41, W74, W358, W420 and F227) lining up the channel interior, forms the ‘greasy slide’, and actively participate in carbohydrate (maltooligosaccharides are in apolar van der Waals contact with the “greasy slide”) transport. Tyrosine 118 (Y118), located opposite of the greasy slide has a major impact on ion and carbohydrate transport through LamB [54,55]. When all six residues of the greasy slide are mutated to alanine, the mutation Y118W is sufficient to confer to LamB maltopentaose transport *in vivo* and maltopentaose binding *in vitro*. How the mutation in LamB could correlate with the resistance to phage λ infection? We have summarized the mutants of LamB that conferred resistance for phage λ in Table 1 [56]. Only about half of the phage λ resistant mutants are surface exposed, and are labeled in yellow in Figure 2D [54]. The remaining mutations (labeled in red in Figure 2D) might have indirect effect. They may alter or the cause structural change on the surface, or they might alter the dynamic behavior of the loops. For instance, one mutation, G18V, is known to affects the stability of trimers and may thus have long‑range effects [56]. Similar to the LamB receptor, a number of studies had been carried to understand how λ utilizes the LamB receptor to inject its DNA inside the host cell [24,25]. Phage λ tail contains a hollow tube that consists of 32-stacked disks, where each disk is formed by six subunits of the major tail protein gpV, arranged such that each disk has a central, 3 nm hole. Phage λ uses this channel to eject its DNA, though the tail by itself (without the head) can attach to the host cell. Genetic evidence indicates that gpJ directly interacts with the outer membrane protein LamB during the attachment of the bacteriophage to the surface of the cell. When the J gene from phage λ was substituted with the tail fiber gene from a closely related bacteriophage 434, the resulting phage was found to bind to a different membrane receptor OmpC [16], which phage 434 uses for infection [57]. Further studies indicate that the C-terminal of gpJ protein determines the host specificity of the phage [24,25]. Electron microscopy imaging of soluble LamB receptor and LamB protein incorporated into liposomes revealed the presence of two different types of bacteriophage λ—LamB complexes. In one type of the complexes binding occurs near the end of the tail fiber (J protein), while in the other type of complexes the distal end of the tail tube was directly and irreversibly attached to the receptor particles or the liposome. Genetic studies showed that the mutations in λ phages that have compensatory effect with the LamB mutants tightly blocks the phage λ adsorption are located in the C‑terminal portions of J [24]. In fact, using a chimeric protein comprising the last 249 amino acids of J in fusion to the C-terminal end of the carrier maltose binding protein (MBP) could bind to LamB trimers and inhibited recognition by anti-LamB antibody [17]. Electron microscopy study showed that this chimeric J protein could also bind to the LamB at the cell surface and this interaction prevented λ adsorption. In this same study, when this chimeric protein was reconstituted with either LamB or the loop deletion mutant LamB 4+6+9v, both of them showed similar blocking of ion current in the lipid bilayer experiment, which indicated that the phage λ binding includes not only the extracellular loops.

**Table 1:** Mutation in LamB receptor that confers resistance to lambda phage infection (From [56]).

Residue(s)	Substitution
18	Gly → Val
148	Glu → Lys
151	Gly → Asp
152	Ser → Phe
154	Ser → Phe
155	Phe → Ser
163	Tyr → Asp
164	Thr → Pro
245	Gly → Arg
245	Gly → Val
247	Ser → Leu
249	Gly → Asp
250	Ser → Phe
259	Phe → Val
259	Phe → Val
382	Gly → Asp
382	Gly → Val
401	Gly → Asp

## 3. The λ Phage LamB Target Finding Process

Numerous genetic and biochemical studies had been carried out to provide the nature of interaction of the LamB receptor and protein J. Recently, a study of the spatiotemporal dynamics by which a phage λ, initially diffusing in bulk, arrives at a specific LamB site on the cell surface, provided a fundamental understanding of the initial viral-host target finding process.

The classical problem of diffusion limited target finding on a cell surface was first addressed by the concept of reaction rate enhancement by dimensional reduction (RREDR), introduced by Adam and Delbrück [58]. To explain the enhancement of adsorption rates they proposed a two-stage capture model , where free phages diffuse in three dimensions (3D), but once in contact with the cell surface this 3D motion is replaced by a two-dimensional (2D) ‘random walk’ on the cell surface, until it is captured by the receptor. This model of reduction in spatial dimension, from 3D to 2D, implementing a 3D+2D searching strategy initially explained the accelerated rate of the process of target finding. In later years, Berg and Purcell showed that to make this 2D diffusion to be advantageous, the adsorption energy has to be strong enough to keep the phage on the surface of the bacterial cell while weak enough to allow the 2D diffusion [59]. Nonetheless, this model was also the first to predict the adsorption rate k as a function of receptors N on a bacterium and the calculated maximum adsorption rate (k_max_) based on this model was noticeably less than experimental findings, which leads to hypothesize the possible contribution of bacterial swimming to the search process. Further understanding of the adsorption process comes from a recent work [60], which showed that upon incubation of phage λ with *E. coli* strain Ymel (this strain carries wild type λ receptor) in a solution of 10 mM MgSO_4_ (pH = 7.4), the decreases of population of free phage in bulk obeys a double‑exponential function with a fast and a slow decay time. Both the fast and slow processes are specific to interactions between phage λ and its receptor and henceforth the interaction is an on-and-off process followed by an irreversible binding. It is also noteworthy that the reversible and the irreversible binding rate in nearly independent of temperature, suggesting the entropic nature of phage retention by the receptor. However, this simplified model is based on uniform distribution of receptor sites in the cell surface of the bacteria, while studies using fluorescently labeled λ tail showed that spatial distribution of LamB in the outer membrane is not uniform, and rather LamB accumulated in irregular and spiral patterns that are dynamic and depend on cell length [61]. In another study, fluorescently labeled λ phages adsorbed on individual cells revealed a preferential binding of phages to the bacterial poles rather than cover the cell surface uniformly [62]. Interestingly, this preference for polar sites is not restricted to λ. When similar experiments were carried with other *E. coli* phages such as P1 [63], virulent coliphages T4 [64] and T7 [65] or with other bacteria than *E. coli* K-12, like *Yersinia pseudotuberculosis* (and with T7-like Yersinia phage A1122) [66] and *Vibrio Cholera* (with T4 like vibrio phage KVP40) [67], similar preference to bacterial poles had been documented (See Table 2) [62]. Taken together, these findings indicated that a model based on uniform 2D diffusion search process is lacking. 

**Table 2:** Localization of different phages on cell surface. QDots label phages are compared for their localization on cell surface (Adopted and modified from [62]).

Phage: Host	Foci at the pole and mid-cell (%)	Foci in other location (%)
λ: *E. coli*	69	31
T7: *E. coli*	71	29
P1: *E. coli*	78	22
T4: *E. coli*	95	5
λØ80: *E. coli*	68	32
KVP40: *V. cholera*	73	27
ØA1122: *Y. pseudotuberculosis*	68	32

**Figure 3:**
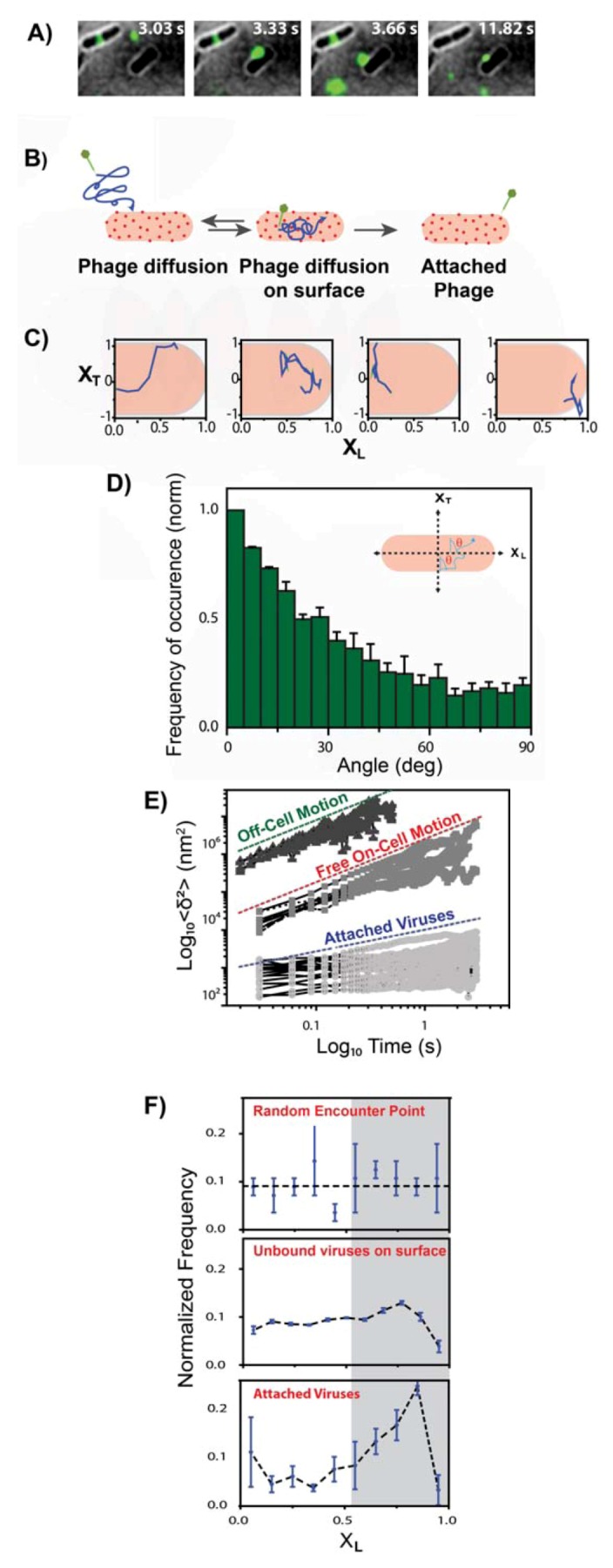
The target-finding process of individual viruses. (**A**) Time-lapse images of a single fluorescently label λ-phage virus (green spot) diffusing near and on an *E. coli* host cell (black) (images scale: height = 10 µm, width = 11 µm). (**B**) Cartoon of the observed stages for virus receptor-finding process: (I) Virus initially diffuses freely until it encounters a cell, followed by (II) motion on the host cell and (III) binding to a receptor (or detachment from the host cell and continued free diffusion). (**C**) Four representative single‑virus trajectories plotted in normalized bacterial coordinates (X_L_, long axis: length normalized coordinate; X_T_, short axis: width normalized coordinate [26]), showing a tendency for motion along the short axis X_T_ (perpendicular to the long axis). (**D**) Normalized distribution of the angle between the momentary displacement vector and the short axis, X_T_, (138 viral trajectories). The histogram shows a predominant inclination for motion along the short axis (X_T_) with a mean angle = 29.6 ± 0.4° (mean ± SE, SD = 24.38 degrees). (**E**) The calculated MSD as a function of lag time for individual viral trajectories in each of the observed regimes, forming distinct groups with more than an order of magnitude separation in MSD values: off-cell diffusion trajectories (green), on‑cell diffusion (red), and attachment (blue). Trajectories for both off-cell diffusion and on-cell diffusion yielded a log-log slope of ~1, indicative of normal diffusion. Viruses bound to the host showed either small, confined movement or no movement, with a 0.5–0 slope range. (**F**) Distributions of virus positions along the cell (X_L_) throughout the spatial focusing process. Error bars: mean ± SE. (i) Initial random point of encounter (top panel, 47 viral trajectories). (ii) Unbound viruses moving on the surface of the host (middle panel, 138 viral trajectories). (iii) Distribution of final infection sites with a clear trend for polar localization (bottom panel, 59 viral trajectories). The area of all three distributions was normalized to facilitate comparison between the different distributions. Adopted and modified after [26].

An accurate depiction of the search process was recently established using single-particle tracking experiments of fluorescently labeled phages. This experimental approach allowed to monitor the early stages of infection in live bacteria at single-virus:single-cell level with nanometer localization accuracy and ~30-ms time resolution [26]. In this study live *E. coli* cells were first attached to the surface of a microfluidic chamber followed by addition of fluorescent viruses enabling to monitor their adsorption onto cells. The motion of individual phages was categorized in three different modes, free diffusion, motion on the surface of the host cells, and attachment (Figure 3A,B). Similar to the classical pictures [58], free phages initial diffuse in 3D until they encounter a bacterial cell, hence their motion will transition into a 2D diffusion on the cell surface. The phages will continue to diffuse on the surface of the cell until they either irreversibly bind to a receptor, or fall off and continue their free 3D motion. It is noteworthy that the diffusion coefficient value for each mode of motion is strikingly different (Figure 3D). Both free viruses and viruses moving on the cell surface followed normal Fick’s laws of diffusion but with an order of magnitude difference in their diffusion coefficient. On the other hand, viruses that were attached to the cells exhibited slow local motion. However, in contrary to the classic view of uniform 2D movement of phages on the cell surface, phages exhibited a distinctly anisotropic motion pattern, with a tendency to move along the short axis of the cell (Figure 3C). Their 2D motion on cell surface was also spatially heterogeneous, showed a spatial focusing along the cell. The bound phages also showed a distinct preference for the poles of the cells (Figure 3F). 

**Figure 4:**
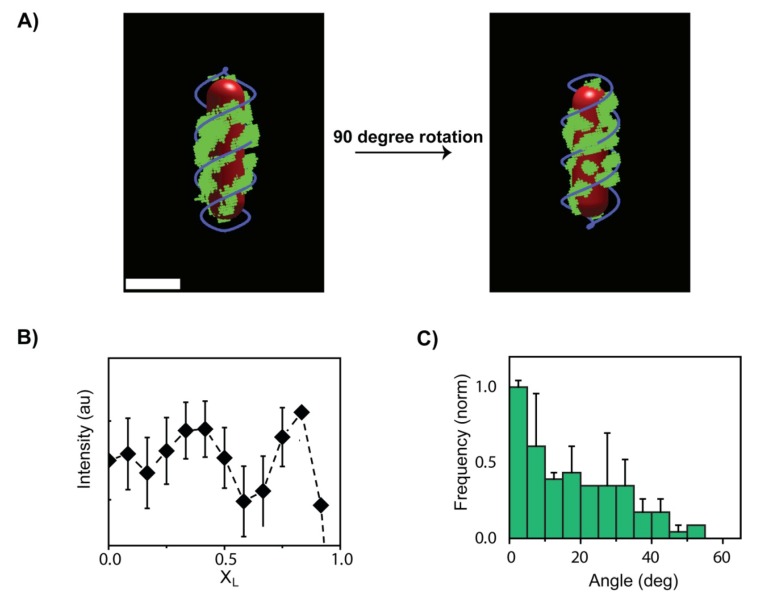
Arrangement of the LamB receptors network on cell surface. (**A**) One cell is shown. Rendered 3D image obtained by sectioning epifluorescence microscopy of cells with labeled receptors, showing nearly continuous helices (scale bar = 2 μm). Quantum dots (green) were used to label the receptor and cell outline is in red. The pattern is described by two helices in blue. (**B**) Normalized LamB distribution along X_L_ averaged for 50 cells. A clear peak at the cellular poles indicates of a high concentration of receptors. (**C**) Angular distribution of LamB bands from 98 cells, showing a tendency for band orientation along the short axis X_T_ (perpendicular to the long axis). Adopted and modified after [26].

**Figure 5:**
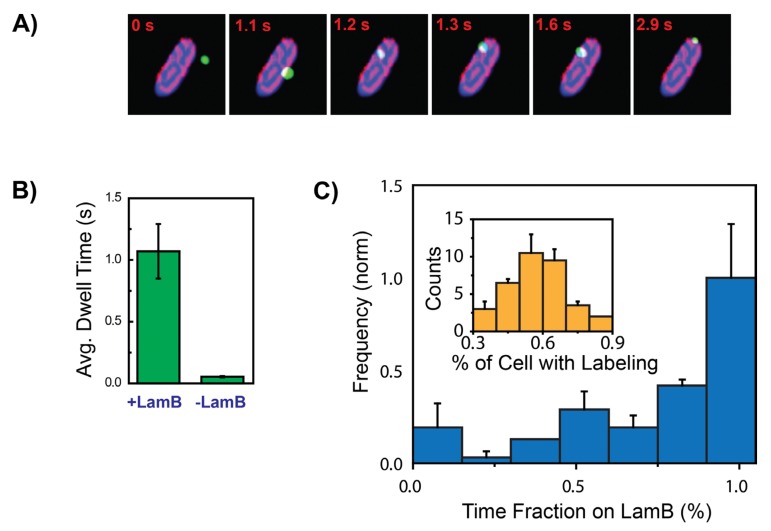
Viral motion on the cell surface is dominated by the interaction with LamB receptors. (**A**) Time-lapse images of the colocalization of a free virus (green) moving on a cell (blue) with LamB receptor bands (purple). The virus is observed to be predominantly moving on the LamB receptor bands (scale bar = 2 µm). (**B**) The mean dwell times of viruses on host cell with (+LamB) and without (−LamB) LamB. (**C**) Colocalizationdwell‑time analysis of 70 unbound viruses moving on cells with labeled bio LamB, showing the distribution of the fraction of a trajectory the viruses spent on LamB regions, with strong prevalence for residing in highly labeled, receptor-rich regions. Inset: Distribution of the cell area (fraction of entire area) with labeled receptors. Adopted and modified after [26].

These observations were hypothesized to be linked to an ordered pattern of phage receptors, LamB, on the surface of the cell. This hypothesis is also in good agreement with the previous results [61] which showed, using fluorescently labeled phage tails that a spatial arrangement of LamB receptors on the *E. coli* surface was reminiscent of the helices and rings found for other bacterial surface proteins [68,69]. To test that notion, the arrangement of LamB receptors on the cell surface was examined using an *E. coli* strain (S2188:pLO16) with an inducible expression for a modified biotinylated version of the LamB protein [70], enabling to specifically label LamB receptors using SA‑conjugated fluorophores. Labeling multiple receptors with a high concentration of QDs (10 nM) resolved the spatial organization of LamB on the cell surface, clearly showing various striped patterns reminiscent of rings and helices (Figure 4A). The distribution of receptors along individual cells showed distinct peaks corresponding to the observed rings and helices with high receptor concentration around the cell pole. The striking resemblance of organization of the LamB receptors with the features exhibited by viruses moving on the cell surface, suggest a unique virus-receptor interaction resulting in an increased viral residence in receptor rich regions. This idea is further supported by: (1) a similar angular distribution for viral trajectories and LamB bands (Figure 4C), (2) an increased viral affinity for polar localization, and (3) distinct LamB bands at poles (Figure 4B). Further proof that the viral motion of phages on the cell surface is governed by interaction with LamB receptors was provided by comparing the dwell times of viruses on wild-type cells and receptor-deleted cells (Figure 5B) [70,71]. Cells lacking receptors exhibited a >15-fold decrease in dwell time, indicating that LamB receptors are required for prolonged interaction between viruses and cell surface. Along this line of evidence, co localization experiments of moving viruses and receptors (Figure 5A,C) showed that the viruses spent a mean of 73.6 ± 3.7% of their total trajectory time in receptor-rich regions.

## 4. Theoretical Model for Viral Target Finding

**Figure 6:**
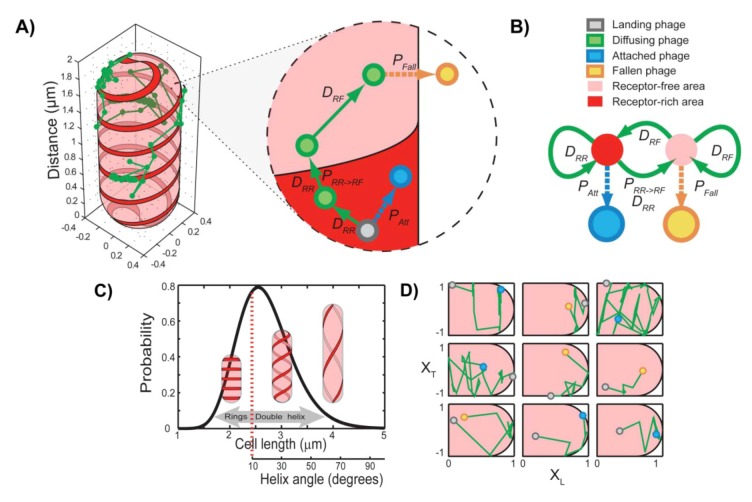
Theoretical model of phage target finding. (**A**) A schematic description of the model, showing a typical phage trajectory (green) on the surface of an *E. coli *cell. Cell shape was modeled as a cylinder with hemispherical caps (radius R_c_ = 0.4 μm). The cell surface was divided into receptor-free (transparent pink) and receptor-rich (solid red) areas. The receptors form a 50-nm-thick double helical or multi-ring pattern along the cell surface. The zoomed-in area shows the kinetic scheme in detail. A phage located in a receptor-rich zone can either move within the receptor area with a stepsize consistent with the diffusion coefficient D_RR_, become attached with a probability P*_Att_* (chosen to match the experimentally observed dwell time to attachment), or move into a receptor-free zone. This move can be rejected with a probability 1 − P*_NR __→ R_*, in which case the move will be repeated. On the other hand, a phage located in a receptor-free area can move with a diffusion coefficient D_NR_. If the phage attempts to enter the receptor zone, the move will always be allowed. The phage can also fall off the cell with a probability P_Fall_ = τsim/<τ*_Fall_>*, where τ*_sim_* is the time step of the simulation and <τ*_Fall_>*, is the experimentally observed dwell time to fall-off in a LamB-*E. coli* strain. (**B**) A simplified kinetic scheme of the model, shown as a four-state, discrete-time Markov chain. The nonreceptor (NR) and receptor (R) states are transient, and the attachment (A) and fall-off (F) states are absorbing. (**C**) Generating population heterogeneity. Cell lengths were randomly chosen from a log-normal distribution spanning the experimentally observed cell sizes. The cell size defines the pitch of receptor double helix according to the relationship obtained experimentally. For short cells with helix angles of <10, a multi-ring pattern was used instead of a double helix. (**D**) Representative 2D projections of phage trajectories in normalized units (green lines). The initial landing site is shown as a gray circle. Panels in the first two rows display trajectories ending in attachment (blue circle), and panels in the last two rows display trajectories ending with the phage falling off the cell (at a position denoted with a yellow circle). Adopted and modified after [26].

A quantitative understanding of the dynamics of viral receptor-finding process came from a theoretical model obtained by simulating the motion of 10,000 viruses using an algorithm for curved surface diffusion (Figure 6) [26,72,73]. In this model, *E. coli* cells were considered as cylinders with spherical caps. For each cell, the cell size defined the pitch of a double helix. This double helix was defined as a receptor-rich area, and the rest of the cell surface was defined as receptor-free. A virus located in a receptor-rich zone may diffuse randomly within the receptor-rich area, it may become attached to the surface, or it may move into a receptor-free area, whereas a virus located in the receptor-free area may diffuse within this area, diffuse into the receptor-rich area, or fall off the cell surface. The resulting spatial focusing features generated by the model and the resulting trajectories showed to be in agreement with the experiments. Viruses arriving at random places along the cell followed by gradually concentrate at receptor-rich regions and the final attachment sites show a pronounced preference for the receptor-rich areas, and especially the cell poles. 

## 5. Summary

The mechanisms by which molecules, viruses and cells find their targets in biological systems are crucial for a fundamental understanding all biological processes. The interaction of bacteriophage λ with its cell surface receptor, LamB, and its receptor finding process serves an excellent model for this purpose. A plethora of theoretical and experimental studies had been done to address this problem [14,15,16,18,19,20,21,22,23,24,25,26]. The classic RREDR model [58], a reduction of dimensionality from 3D motion in bulk to 2D motion on cell surface, was initially considered to be in good agreement with the experimental observation [14,15,58,59,60,74]. However, finding that the interaction of viruses with a localized network of receptors on the cell surface limits their sampling motion to a fraction of the surface, in effect rendering it quasi-one-dimensional, indicates that the searching process is more complex than what was previously thought and paved the way for further modification of the existing model. We are concluding our present discussion by speculating that the connection between target finding processes and the spatial distribution of the target may be universal, and could be better addressed by considering fractal dimensional motion. 
